# Changes in intracellular copper concentration and copper-regulating gene expression after PC12 differentiation into neurons

**DOI:** 10.1038/srep33007

**Published:** 2016-09-13

**Authors:** Yasumitsu Ogra, Aya Tejima, Naohiro Hatakeyama, Moeko Shiraiwa, Siyuan Wu, Tsutomu Ishikawa, Ayako Yawata, Yasumi Anan, Noriyuki Suzuki

**Affiliations:** 1 Laboratory of Toxicology and Environmental Health, Graduate School of Pharmaceutical Sciences, Chiba University, Chuo, Chiba 260-8675, Japan; 2 Laboratory of Chemical Toxicology and Environmental Health, Showa Pharmaceutical University, Machida, Tokyo 194-8543, Japan; 3 Department of Medicinal Organic Chemistry, Graduate School of Pharmaceutical Sciences, Chiba University, Chuo, Chiba 260-8675, Japan

## Abstract

It is suspected that some neurodegenerative diseases are a result of the disturbance of copper (Cu) homeostasis, although it remains unclear whether the disturbance of Cu homeostasis has aberrant effects on neurons. Herein, we investigated Cu metabolism specifically in neurons in terms of changes in the intracellular Cu concentration and the expression of Cu-regulating genes, such as Cu transporters and metallothioneins (MTs), before and after the differentiation of rat pheochromocytoma cells (PC12 cells) into neurons. After the differentiation, Cu and Zn imaging with fluorescent probes revealed an increase in intracellular Cu concentration. The concentrations of other essential metals, which were determined by an inductively coupled plasma mass spectrometer, were not altered. The mRNA expression of the Cu influx transporter, Ctr1, was decreased after the differentiation, and the differentiated cells acquired tolerance to Cu and cisplatin, another substrate of Ctr1. In addition, the expression of MT-3, a brain-specific isoform, was increased, contrary to the decreased expression of MT-1 and MT-2. Taken together, the differentiation of PC12 cells into neurons induced MT-3 expression, thereby resulting in intracellular Cu accumulation. The decrease in Ctr1 expression was assumed to be a response aimed at abolishing the physiological accumulation of Cu after the differentiation.

Copper (Cu) exerts ambivalent effects on living organisms. It is an essential metal at physiological concentrations but shows severe toxicity when its concentration exceeds the physiological range. As an essential metal, Cu is used in respiration, and is required as a cofactor of redox-regulating enzymes, such as superoxide dismutase (Sod1), ceruloplasmin, lysyl oxidase, tyrosinase, and dopamine β-hydroxylase^1^,^2^. To act as a cofactor, Cu in the body exists in the mono-(cuprous, Cu^+^) or divalent (cupric, Cu^2+^) form. The transition between the two oxidation states readily generates reactive oxygen species (ROS). Thus, the influx, efflux, and intracellular distribution of Cu at the fixed oxidation state are strictly regulated. Several groups of Cu-regulating proteins have been identified in mammalian cells. The first group consists of Cu transporters that transport Cu across the plasma membrane. Ctr1 (copper transporter 1) encoded by *SLC31A1* gene is an integral membrane protein that is structurally and functionally conserved from yeast to human, and is a high-affinity importer of Cu into eukaryotic cells^3^. Cu-transporting P-type ATPases, *i.e*., Atp7a and Atp7b, are expressed on the Golgi apparatus and participate in the efflux of Cu from cells. Atp7a is expressed in all tissues except liver, whereas Atp7b is expressed primarily in the liver^4^. The second group consists of intracellular Cu delivery proteins, or the so-called “Cu chaperones.” Cu transported by Ctr1 associates with one of three Cu chaperones, Atox1 (antioxidant protein 1), Cox17 (cytochrome *c* oxidase copper chaperone), or Ccs (copper chaperone for Sod1), to be escorted to organelles or cuproenzymes in cytoplasm. First, Atox1 hands over Cu to Atp7a and Atp7b expressed on the surface of the Golgi apparatus^5^. Second, Cox17 loads Cu to cytochrome *c* oxidase (Cco) via SCO1 (synthesis of cytochrome c oxidase) and Cox11, which are Cu recipient proteins on the mitochondrial inner membrane^6^. Third, Ccs delivers Cu to Sod1 in cytosol by forming a heterodimer between itself and Sod1^7^. The third group of Cu-regulating proteins is composed of metallothioneins (MTs). MTs are cytosolic proteins that bind excess intracellular Cu via Cu-thiolate clusters to mask Cu toxicity^8^. Four main isoforms are expressed in mammals: MT-1, MT-2, MT-3, and MT-4^9^,^10^. MT-1 and MT-2, called classic MTs, are ubiquitously expressed in all cell types, whereas, MT-3 and MT-4 are tissue-specific. In particular, MT-3 is specifically expressed in brain and exhibits enzyme activity as a growth inhibitory factor of neurons^11^. The fourth group includes a novel Cu-regulating protein that was recently characterized, *i.e*., the Cu metabolism gene Murr1 (mouse U2af1-rs1 region 1) domain (Commd1)^12^,^13^. However, little is known about the actual role of Commd1^14^,^15^.

It has been suggested that certain neurodegenerative diseases, such as amyotrophic lateral sclerosis (ALS), Alzheimer’s disease, and prion diseases, are a result of the disturbance of Cu homeostasis^16^,^17^,^18^. A defect in chromosome 21, which codes for sod1, is associated with approximately 20% of familial cases of ALS, or approximately 2% of all ALS cases^19^,^20^. It is shown that Cu ion enhances the formation of amyloid β plaques, which results in the progression of Alzheimer’s disease, and accumulates in the amyloid β plaques at a high concentration^21^,^22^. It is also suggested that the disrupted regulation of copper ions in the brain is a key factor in Creutzfeldt-Jakob disease and other prion diseases^23^. In addition, a significant decrease in Cu concentration in degenerating substantia nigra in Parkinson’s disease patients has been reported^24^. Those experimental results have motivated us to speculate that neurons have a unique mechanism for maintaining Cu homeostasis compared with other types of cells.

PC12 cells are pheochromocytoma cells originating in rat adrenal medulla. PC12 cells can differentiate into neurons by treatment with nerve growth factor (NGF)^25^. Hence, PC12 cells seem to be a useful model to observe specific Cu metabolism during the process of differentiation into neurons. Indeed, Cu concentration in PC12 cells was increased by NGF treatment^26^. In this study, we evaluated intracellular metal concentration and distribution to clarify whether the increase in intracellular metal concentration was specific to Cu or not. We also determined the expression of Cu-regulating genes before and after the differentiation of PC12 cells into neurons, to clarify the mechanisms underlying the increase in intracellular Cu concentration.

## Results

### Generation of neurite-bearing PC12 cells after treatment with NGF

**Figure 1:**
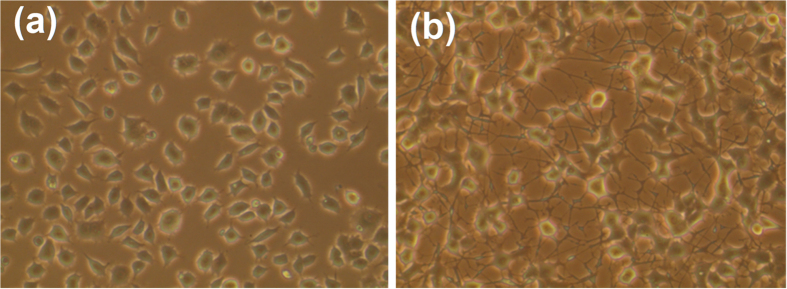
Phase-contrast photomicrographs and NeuN expression in PC12 cells not treated with (**a**) or treated with (**b**) NGF. PC12 cells differentiated into neurons after treatment with 50 ng/mL NGF for six days.

Naive PC12 cells, which are pheochromocytoma cells originating in rat adrenal medulla, generated neurites after treatment with 50 ng/mL NGF for six days (Fig. 1a,b). Hence, the naive PC12 cells were able to differentiate under the conditions we adopted. The conditions for PC12 cell differentiation were used in the following experiments.

### Metal distribution and concentration

**Figure 2:**
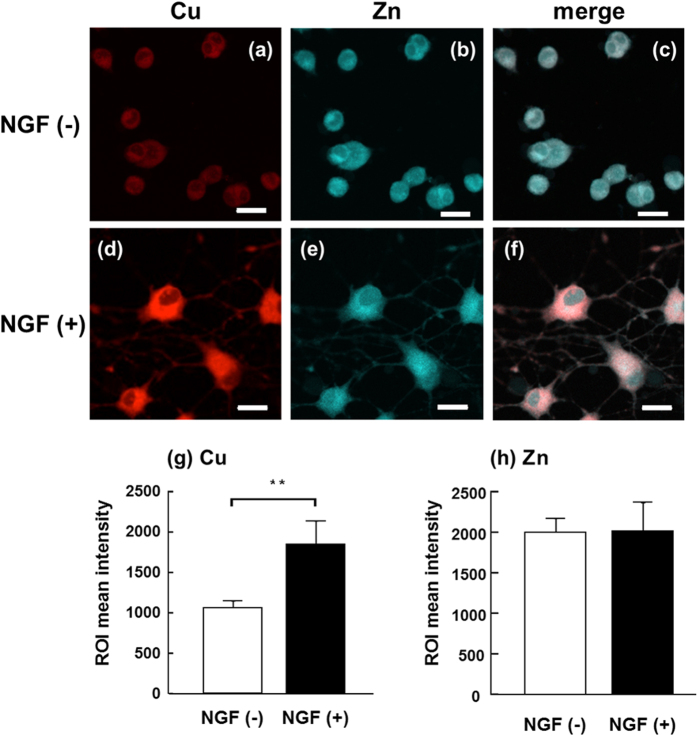
Fluorescence images of naive and differentiated PC12 cells treated with CS1 and Zinquin. Naive (**a–c**) and differentiated (**d–f**) PC12 cells were doubly stained with 5 μM CS1 (**a,d**) for 5 min and 5 μM Zinquin (**b,e**) for 30 min at 25 °C in PBS. Superimposed images are also presented (**c,f**). Scale bar indicates 20 μm. The fluorescence intensities of Cu^+^-CS1 (**g**) and Zn^2+^-Zinquin (**h**) complexes were quantitatively analyzed with software installed in our confocal laser microscope. Values are expressed as means ± S.D. of three independent experiments. The difference at the level of significance of *p* < 0.01 between the naive (NGF(−)) cells and the differentiated (NGF(+)) cells is indicated by **.

The fluorescence of the Cu-CS1 complex was preferably detected in the cytoplasm of naive PC12 cells (Fig. 2a). The Zn-Zinquin complex was also detected in the cytoplasm (Fig. 2b). In the differentiated cells, the fluorescence intensity of the Cu-CS1 complex seemed to increase, whereas that of the Zn-Zinquin complex was not altered (Fig. 2d,e). Neurites of the differentiated cells were stained with CS1 and Zinquin, suggesting that Cu^+^ and Zn^2+^ were distributed to the neurites. The Zn-Zinquin complex was weakly detected in the nuclei of the differentiated cells, and the Cu-CS1 complex was more specifically distributed to the cytoplasm than the Zn-Zinquin complex (Fig. 2e,f). Although the fluorescence intensity of the Zn-Zinquin complex did not change, that of the Cu-CS1 complex was significantly increased by the differentiation (Fig. 2g,h).

**Figure 3:**
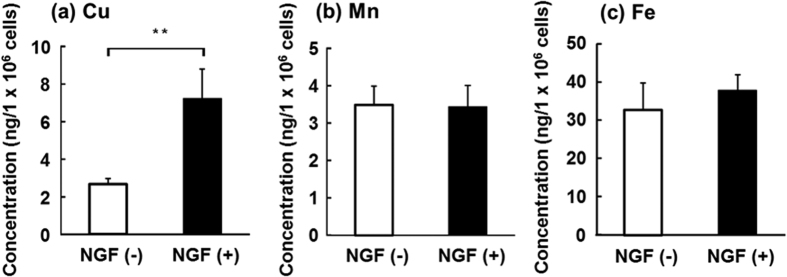
Effect of NGF treatment on the concentrations of essential metals in PC12 cells. PC12 cells differentiated into neurons after treatment with 50 ng/mL NGF for six days. The concentrations of Cu (**a**), Mn (**b**), and Fe (**c**) in the naive (open columns) and differentiated (closed columns) PC12 cells were quantified by ICP-MS at *m*/*z* 65, 55, and 57, respectively. Values are expressed as means ± S.D. of three independent experiments. The difference at the level of significance of *p* < 0.01 between the naive (NGF(−)) cells and the differentiated (NGF(+)) cells is indicated by **.

As the appropriate fluorescent probes for other essential metals, such as Mn and Fe, were not commercially available, changes in the concentrations of those metals after the differentiation were quantified by ICP-MS. As observed by confocal laser microscopy (Fig. 2a,d), Cu concentration was significantly increased by the differentiation (Fig. 3a). In contrast, no significant changes in the concentrations of Mn and Fe were induced by the differentiation (Fig. 3b,c). This suggests that the differentiated cells specifically accumulated Cu among the essential transition metals.

### Changes in mRNA expression of Cu-regulating genes

**Figure 4:**
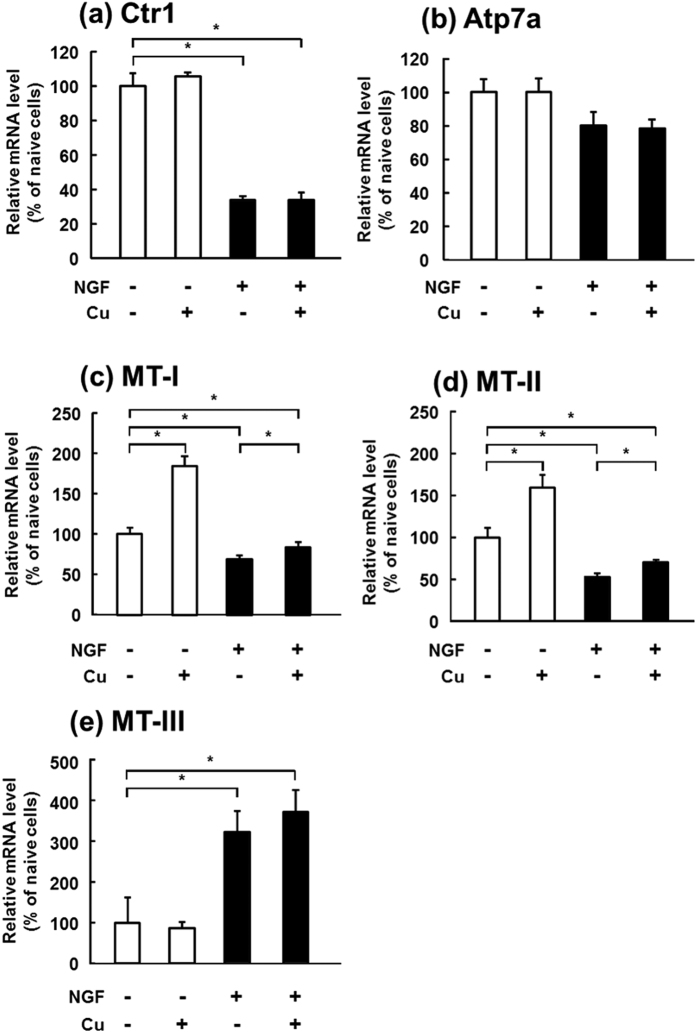
Changes in mRNA expression of Cu-regulating genes after treatment of PC12 cells with NGF and Cu(I). PC12 cells differentiated into neurons after treatment with 50 ng/mL NGF for six days. Differentiated (NGF(+)) cells and naive (NGF(−)) cells were exposed to 10 μM copper acetate plus 30 μM GSH for 6 h in the differentiation medium. mRNA expression of Ctr1 (**a**), Atp7a (**b**), MT-1 (**c**), MT-2 (**d**), and MT-3 (**e**) in the naive (open columns) and differentiated (closed columns) PC12 cells was determined by real-time PCR. Values are expressed as means ± S.D. of three independent experiments. The difference at the level of significance of *p* < 0.05 between the indicated groups is indicated by *.

The mRNA expression of Ctr1, a Cu influx transporter, in the differentiated cells was reduced to 33% of that in the naive PC12 cells (Fig. 4a). Cu addition had no effects on the Ctr1 expression in both naive and differentiated cells. Although the expression of Atp7a, a Cu efflux transporter, tended to decrease in the differentiated cells, the decrease was not significant (Fig. 4b). Cu addition had no effects on the Atp7a expression in both naive and differentiated cells.

MT-1 and MT-2 expressions were significantly decreased by the differentiation (Fig. 4c,d). Although MT-1 and MT-2 expressions were significantly induced by the Cu addition in the naive and differentiated cells, the expression in the differentiated cells was less induced than that in the naive cells. On the other hand, the expression of MT-3, an organ-specific isoform of MTs, was increased to more than 300% of that in the naive cells by the differentiation with or without the Cu addition (Fig. 4e). However, no significant increase in the expression was observed by the Cu addition to the naive cells.

**Figure 5:**
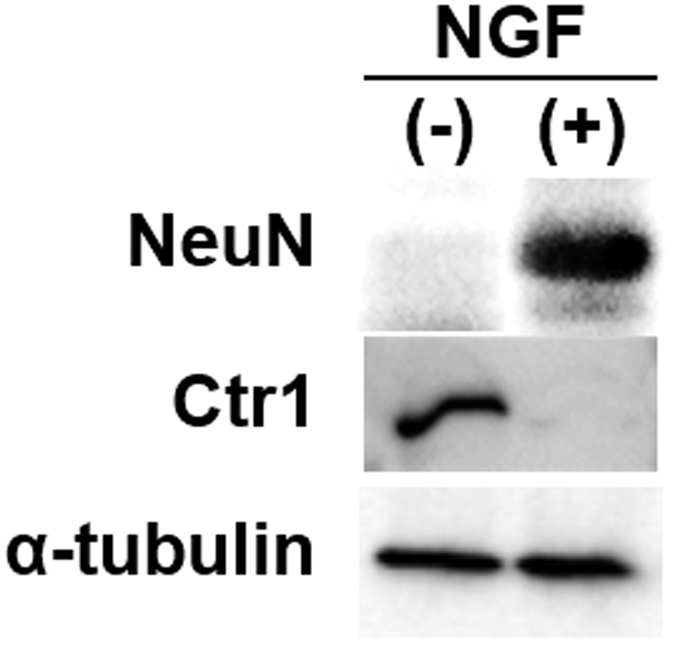
Changes in protein expression after treatment of PC12 cells with NGF. PC12 cells differentiated into neurons after treatment with 50 ng/mL NGF for six days. Protein expression of NeuN (top), Ctr1 (middle), and α-tubulin (bottom) in the naive (NGF(−)) and differentiated (NGF(+)) PC12 cells was analyzed by Western blotting.

Since the mRNA expression of Ctr1 was severely reduced in the differentiated cells, the protein expression of Ctr1 was also evaluated. The protein expression of NeuN, a neuron biomarker, served as a positive control of the differentiation (Fig. 5, top). The protein expression of NeuN was coincident with the morphological changes of the differentiated and naive cells (Fig. 1). Although the protein expression of Ctr1 in the naive cells was detected, no protein expression of Ctr1 was detected in the differentiated cells (Fig. 5, middle). The protein expression of α-tubulin served as an internal standard (Fig. 5, bottom).

### Comparison of tolerance to metal compounds between naive and differentiated cells

**Figure 6:**
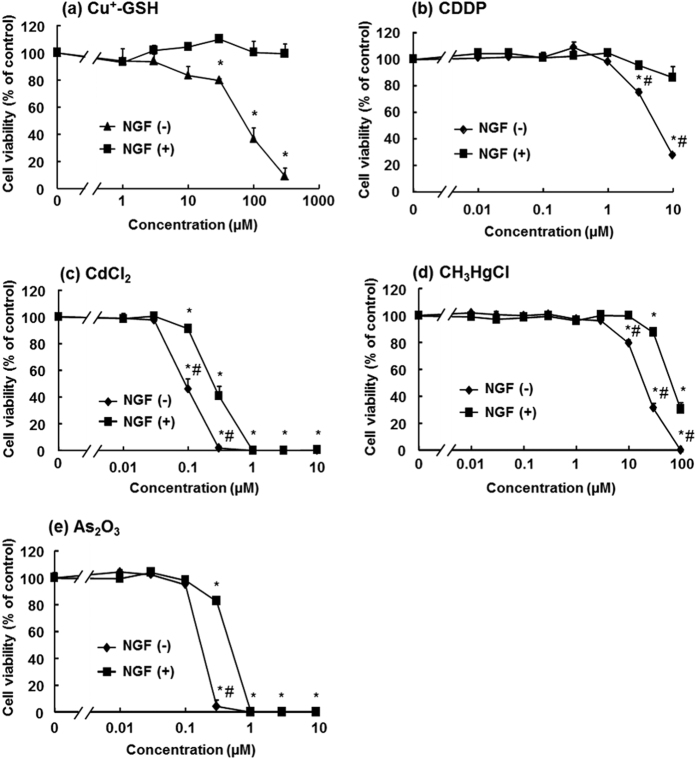
Effect of NGF treatment on the tolerance of PC12 cells to metal compounds. Differentiated (NGF(+)) cells and naive NGF(−)) cells were exposed to 0, 1.0, 3.0, 10, 30, 100, and 300 μM Cu^+^-GSH complex (**a**); 0, 0.01, 0.03, 0.1, 0.3, 1.0, 3.0, and 10 μM cisplatin (**b**); cadmium chloride (**c**); methylmercury chloride (**d**); and 0, 0.01, 0.03, 0.1, 0.3, 1.0, 3.0, 10, 30, and 100 μM arsenous acid (**e**) for 24 h. Cell viability was determined by MTT assay. The differences at the level of significance of *p* < 0.05 between non-treated and treated groups, and naive (NGF(−)) and differentiated (NGF(+)) are indicated by * and ^#^, respectively.

In the naive cells, cell viability was decreased by the Cu treatment in a dose-dependent manner (Fig. 6a). Contrary to the naive cells, the differentiated cells showed tolerance to the Cu treatment, and the tolerance showed a significant difference at 30 μM or higher Cu concentration. The cell viabilities of the naive and differentiated cells treated with cisplatin, another substrate of Ctr1, showed the same tendency as those of cells treated with Cu (Fig. 6b). However, the IC_50_ values of Cu and cisplatin for the differentiated cells could not be calculated because those cells showed tolerance to concentrations exceeding those tested. Then, cell viability was evaluated by using metal compounds that were not a substrate of Ctr1, *i.e*., cadmium chloride, methylmercury chloride, and arsenous acid. The differentiated cells showed significantly higher tolerance to cadmium chloride than the naive cells at the concentration of 0.1 and 0.3 μM. The IC_50_ values of cadmium chloride for the naive and differentiated cells were calculated as 0.16 and 0.51 μM, respectively (Fig. 6c). The differentiated cells also showed significantly higher tolerance to methylmercury chloride, an organometallic compound, than the naive cells at the dose of 0.3 μM. The IC_50_ values of methylmercury chloride for the naive and differentiated cells were calculated as 0.20 and 0.58 μM, respectively (Fig. 6d). In addition, the differentiated cells showed significantly higher tolerance to arsenous acid, a metalloid compound, than the naive cells at the doses of 10, 30, and 100 μM. The IC_50_ values of arsenous acid for the naive and differentiated cells were calculated as 49.5 and 78.1 μM, respectively (Fig. 6e). Together, the results indicated that although the differentiated cells showed higher tolerance to the metal/metalloid-induced toxicities than the naive cells, the differentiated cells acquired much higher tolerance to Ctr1 substrates than to non-Ctr1 substrates.

## Discussion

The results of fluorescence imaging and ICP-MS are concrete evidence that PC12 cells specifically accumulated Cu in cytoplasm. Although intracellular Cu concentration was increased, the mRNA and protein expressions of Ctr1, a Cu influx transporter, was decreased by the differentiation. Indeed, the fact that the differentiated cells showed higher tolerance to Ctr1 substrate than the naive cells. It has been pointed out that Ctr1 is controlled not at the transcriptional level but at the post-translational level depending on the Cu concentration around cells^27^. Ctr1 protein is localized stably on the plasma membrane under the Cu-depleted condition, but loses intracellular stability under the Cu-replete condition^28^. In this study, Crt1 showed decreases in functional and transcriptional activities and hence, it was concluded that the Ctr1 expression was decreased by the differentiation of PC12 cells into neurons at the transcription level. Based on this scenario, a discrepancy would be expected regarding the intracellular Cu concentration: if Ctr1 expression were decreased by the differentiation, intracellular Cu concentration would be decreased. However, as mentioned above, this study and a previous study indicated that the intracellular Cu concentration was increased by the differentiation^26^. Thus, we speculated that during the differentiation process, the PC12 cells initially accumulated Cu and then decreased Ctr1 expression. In other words, the differentiation of PC12 cells induced the Cu accumulation and, with the intent of ameliorating the Cu accumulation, Ctr1 expression in the differentiated cells was decreased. Then we ask, why and how do PC12 cells accumulate Cu during differentiation? We speculate that MT-3 plays a role in the differentiation of PC12 cells into neurons.

Although MT-1 and MT-2 usually contain seven Zn ions each, MT-3 isolated from human and bovine brains contains four Cu ions and three or four Zn ions^29^. This suggests that MT-3 more preferably binds Cu than MT-1 and MT-2 under physiological conditions. Therefore, the increase in MT-3 and the decreases in MT-1 and MT-2 result in the accumulation of Cu in the form bound to MT-3. It is known that MT-1, MT-2, and MT-3 are mainly localized in the cytoplasm under physiological conditions. This coincides with the fact that Cu was mainly accumulated in the cytoplasm (Fig. 2). Then, this physiological accumulation of Cu in the differentiated cells was followed by the Ctr1 transcriptional decrease to suppress the further accumulation of Cu.

MT-1 and MT-2 can reduce the toxicities of not only heavy metals but also organometallic compounds and metalloids because they sequester or scavenge those compounds and ROS generated by those compounds via the abundant sulfhydryl groups of cysteine residues in their molecules^30^,^31^,^32^. The cysteine residues of MT-3 are highly conserved relative to those of MT-1 and MT-2. In fact, MT-3 can sequester and scavenge toxic metals and ROS^33^,^34^. Because MT-3 was induced by the differentiation unlike MT-1 and MT-2, the differentiated cells acquired tolerance to cadmium, methylmercury, and arsenite (Fig. 6c–e). It was reported that MT-3 knockout mice presented with severe neuronal damage after cerebral ischemia compared with the wild type^35^. Hence, MT-3 actually acts as a detoxicant and an anti-oxidant of metal compounds and ROS, respectively^36^,^37^,^38^.

Contrary to the role played by MT-1 and MT-2 as a detoxicant of nucleophiles, such as heavy metals and ROS, it is also reported that MT-1 and MT-2 binding Cu act as a pro-oxidant and are thus harmful to cells; MT-1 and MT-2 produce oxidative stress in the liver of LEC rat, an animal model of Wilson’s disease, which shows abnormal Cu accumulation in the liver^39^. The oxidative stress induces the oxidation of Cu-thiolate bonds in MT, thereby resulting in disulfide formation and the release of Cu(I). Cu(I) generates ROS via the Fenton and Haber-Weiss reactions, and ROS induce further oxidation of the Cu-thiolate clusters. This chain reaction is mediated by MT binding Cu and thus, MT is recognized as a pro-oxidant. Those ambivalent characteristics of MT-1 and MT-2 originate in the Cu-thiolate clusters and thus, those characteristics are expected as well in MT-3 having the Cu-thiolate clusters. Collectively, we speculate that Cu physiologically accumulating and binding to MT-3 in neural cells is one of contributors to the pathogenesis of neurodegenerative diseases, by acting as a pro-oxidant.

It is known that MT-1 and MT-2 can be induced by various factors, including heavy metals, ROS, cytokines, hormones, and γ-irradiation^40^,^41^. The molecular mechanisms underlying the induction have been investigated. For instance, the induction by Zn proceeds via the interaction between the *cis* element known as the metal responsive element (MRE) located in the 5’ untranslated region of MT-1 and MT-2 genes, and the transcription factor MTF-1^42^,^43^. The mechanisms of other inducers were also depicted^40^,^41^. In contrast to the induction of MT-1 and MT-2 by heavy metals, no apparent MREs were found in the promoter/enhancer region of MT-3, and the induction of MT-3 by other inducers was also obscure. It should be clarified in future studies what and how factor(s) induce MT-3 during differentiation.

In conclusion, the differentiation of PC12 cells induced physiological Cu accumulation in the cells. The induction of MT-3 by the differentiation may be the primary trigger for the Cu accumulation. MT-3 seems to play a crucial role in Cu homeostasis in neural cells, and the increase in Cu in the form bound to MT-3 is one of the probable factors for the progression of pathological changes in nerve cells.

## Methods

### Chemicals

The Zn fluorescent probe, Zinquin ethyl ester, and 3-(4,5-dimethyl-2-thiazolyl)-2,5-diphenyl-2H-tetrazolium bromide (MTT) were purchased from Dojindo (Kumamoto, Japan). Mouse NGF, glutathione (GSH), and Dulbecco’s Modified Eagle Medium (DMEM) were purchased from Sigma-Aldrich (St. Louis, MO, USA). The clinically used *cis*-diamminedichloroplatinum(II) (cisplatin) solution, Randa^®^ injection, was purchased from Nippon Kayaku Co., Ltd. (Tokyo, Japan). Copper acetate, cadmium chloride, arsenous acid, methylmercury chloride, and other chemicals were purchased from Wako Pure Chemical Industries (Osaka, Japan).

### Cell culture and induction of PC12 cell differentiation

Pheochromocytoma cells originating in rat adrenal medulla, PC12, were obtained from RIKEN Cell Bank (Tsukuba, Japan). The PC12 cells were grown and maintained in DMEM supplemented with 10% fetal bovine serum (FBS; Hana-Nesco Bio, Tokyo, Japan), 10% horse serum (HS; Culture Biologicals, Long Beach, CA, USA), 100 U/mL penicillin (Invitrogen, Carlsbad, CA, USA), and 100 μg/mL streptomycin (Invitrogen) at 37 °C under 5% CO_2_ atmosphere. The cells were passaged before reaching confluence using 0.025% (v/v) trypsin/EDTA in PBS. For the differentiation of PC12 cells, the cells were seeded on a collagen-coated dish at a density of 2.0 × 10^4^ cells/cm^2^. Then, the cells were exposed to 50 ng/mL NGF in medium containing 1% HS for 24 h after seeding in the maintenance medium. The medium was exchanged every two days with fresh DMEM containing 50 ng/mL NGF and 1% HS until six days after the induction of differentiation. The cells cultured in the differentiation medium containing 1% HS without the NGF treatment served as control and are called naive cells in this study.

### Metal imaging

Synthesis of the Cu fluorescent probe, coppersensor-1 (CS1), was achieved by following the same procedure as that reported by Zeng *et al*.^44^. PC12 cells were seeded on a glass-bottom culture dish coated with collagen at 2.0 × 10^5^ cells/dish, and were pre-incubated for 24 h. The pre-incubated cells differentiated into neurons as mentioned above. Both differentiated and naive (non-differentiated) cells were treated with 5 μM Zinquin dissolved in dimethyl sulfoxide for 25 min, and then treated with 5 μM CS1 dissolved in dimethyl sulfoxide for 5 min at 25 °C in PBS. The fluorescence of Cu^+^-CS1 and Zn^2+^-Zinquin complexes in the cells was detected by confocal laser microscopy (A1 confocal laser microscope system, Nikon, Tokyo, Japan) at emission wavelengths of 560 and 490 nm, respectively. The fluorescence intensities of Cu^+^-CS1 and Zn^2+^-Zinquin complexes were quantitatively analyzed with software installed in our confocal laser microscope.

### Determination of metal concentration

The intracellular concentrations of other essential metals having no specific fluorescent probes, namely, manganese (Mn) and iron (Fe), were determined by an inductively coupled plasma mass spectrometer (ICP-MS, Agilent 7700; Agilent Technologies, Hachioji, Japan). Both differentiated and naive cells were collected and wet-ashed with nitric acid (analytical grade; Wako Pure Chemical Industries) to obtain samples for metal determination. The concentrations of Mn, Fe, and Cu were measured by ICP-MS at *m*/*z* 55, 57, and 65, respectively.

### Real-time PCR

The NGF-treated cells and the naive cells were exposed to 10 μM copper acetate plus 30 μM GSH for 6 h in the differentiation medium. Then, total cellular RNA was extracted from the cells using an RNAqueous^®^-Micro Kit (Ambion, Foster City, CA, USA). Ctr1, Atp7a, MT-1, MT-2, and MT-3 mRNA expression was determined by real-time PCR. cDNA was synthesized from 0.5 μg of total RNA with a High Capacity cDNA Reverse Transcription Kit (Life Technologies, Waltham, MA, USA). Amplification reactions were performed with 70 nM each of the forward and reverse primers for Ctr1 (5′-TTGGCTTTAAGAATGTGGACC-3′ and 5′-CATAAGGATGGTTCCATTTGG-3′), Atp7a (5′-TAGACGGCATGCATTGTAAATC-3′ and 5′-TGGATTTTACACCTGGCTTCTT-3′), MT-1 (5′-GAACTGCAAATGCACCTCCTGC-3′ and 5′-CAAGACTCTGAGTTGGTCCG-3′), MT-2 (5′-AACTGCTCCTGTGCCACAG-3′ and 5′-CTCTTTGCAGATGCAGCC-3′) or MT-3 (5′-CCCTGCAGGATGTGAGAAGT-3′ and 5′-TTTGCTGTGCATGGGATTTA-3′), and 50 ng of cDNA. Samples were analyzed in triplicate in a total volume of 50 μL using Applied Biosystems StepOne^TM^ Real-Time PCR System (Applied Biosystems). β-Actin (5′-TTCTTTGCAGCTCCTTCGTT-3′ and 5′-GAGTCCTTCTGACCCATTCC-3′) was used as the internal control for RNA quantification and for monitoring the efficiency of the reverse transcription. The amount of each gene was expressed as a Ct (threshold cycle) value. The RNA expression of each gene in the differentiated cells relative to that in the naive cells was calculated.

### Measurement of cytotoxicity

PC12 cells were seeded on a 96-well plate and differentiated by the same method as that mentioned above. The differentiated cells and the naive cells were exposed to 0, 1.0, 3.0, 10, 30, 100, and 300 μM Cu^+^-GSH complex; 0, 0.01, 0.03, 0.1, 0.3, 1.0, 3.0, and 10 μM cisplatin, cadmium chloride, and methylmercury chloride; and 0, 0.01, 0.03, 0.1, 0.3, 1.0, 3.0, 10, 30, and 100 μM arsenous acid for 24 h. After the treatment, the culture medium was exchanged with fresh differentiation medium and MTT, and the mixture was incubated for another 4 h at 37 °C. After removing the medium, dimethyl sulfoxide (Wako Pure Chemical Industries) was added to the wells to extract MTT formazan. Absorbance at 560 nm was measured by a microplate reader (Molecular Devices, Tokyo, Japan).

### Western blotting

Neuronal nuclear antigen (NeuN), a commonly used neuron biomarker, Ctr1, and α-tubulin serving as an internal control were detected in both NGF-treated and naive PC12 cells. The cells were collected and lysed in 50 mM Tris-HCl, pH 8.8 by sonication. The lysed cells were mixed with 20% SDS, 20% β-mercaptoethanol (ME), and 50% glycerol. The mixture was electrophoresed through 7.5% polyacrylamide gels and transferred to polyvinylidene difluoride (PVDF) membrane at 20 V for 60 min. The membrane was blocked overnight with 5% skim milk in 25 mM Tris-HCl containing 0.9% NaCl and 0.05% Tween 20, pH 7.5 (TBS-T) at 4 °C. Then, the membrane was incubated with anti-NeuN monoclonal antibody (1:300, Millipore, Tokyo, Japan), anti-Ctr1 (1:1,000, Abcam, Tokyo, Japan), or anti-α-tubulin antibody (1:200, Cell Signaling Technology, Danvers, MA, USA) in TBS-T for 1 h and washed three times with TBS-T. The membrane was incubated with sheep anti-mouse IgG conjugated with horseradish peroxidase (1:3000; GE Healthcare, Tokyo, Japan), a secondary antibody for anti-NeuN and anti-α-tubulin, or sheep anti-rabbit IgG conjugated with horseradish peroxidase (1:3000; GE Healthcare, Tokyo, Japan) for anti-Ctr1 in TBS-T containing 2% skim milk, and washed six times with TBS-T. The blots were detected with SuperSignal^®^West Pico Chemiluminescent Substrate (Thermo Scientific, Waltham, MA, USA) by using an LAS-1000 Plus Lumino Image Analyzer (FUJIFILM, Tokyo, Japan) according to the manufacturer’s instructions.

### Statistics

Data are presented as means ± S.D. Statistical analysis included the one-way analysis of variance followed by the Student’s *t*-test.

## Additional Information

**How to cite this article**: Ogra, Y. *et al*. Changes in intracellular copper concentration and copper-regulating gene expression after PC12 differentiation into neurons. *Sci. Rep*. **6**, 33007; doi: 10.1038/srep33007 (2016).
